# SQUID: transcriptomic structural variation detection from RNA-seq

**DOI:** 10.1186/s13059-018-1421-5

**Published:** 2018-04-12

**Authors:** Cong Ma, Mingfu Shao, Carl Kingsford

**Affiliations:** Computational Biology Department, Carnegie Mellon University, Pittsburgh, 5000 Forbes Ave. PA USA

**Keywords:** Transcriptomic structural variation, RNA-seq, TCGA

## Abstract

Transcripts are frequently modified by structural variations, which lead to fused transcripts of either multiple genes, known as a fusion gene, or a gene and a previously non-transcribed sequence. Detecting these modifications, called transcriptomic structural variations (TSVs), especially in cancer tumor sequencing, is an important and challenging computational problem. We introduce SQUID, a novel algorithm to predict both fusion-gene and non-fusion-gene TSVs accurately from RNA-seq alignments. SQUID unifies both concordant and discordant read alignments into one model and doubles the precision on simulation data compared to other approaches. Using SQUID, we identify novel non-fusion-gene TSVs on TCGA samples.

**Electronic supplementary material:**

The online version of this article (10.1186/s13059-018-1421-5) contains supplementary material, which is available to authorized users.

## Background

Large-scale transcriptome sequence changes are known to be associated with cancer [1, 2]. Those changes are usually a consequence of genomic structural variation (SV). By pulling different genomic regions together or separating one region into pieces, structural variants can potentially cause severe alteration to transcribed or translated products. Transcriptome changes induced by genomic SVs, called transcriptomic structural variants (TSVs), can have a particularly large impact on disease genesis and progression. In some cases, TSVs bring regions from one gene next to regions of another, causing exons from both genes to be transcribed into a single transcript (known as a fusion gene). Domains of the corresponding RNA or proteins can be fused, inducing new functions or causing loss of function, or the transcription or translation levels can be altered, leading to disease states. For example, *BCR*-*ABL1* is a well-known fusion oncogene for chronic myeloid leukemia [3], and the *TMPRSS2*-*ERG* fusion product leads to over-expression of *ERG* and helps triggers prostate cancer [4]. These fusion events are used as biomarkers for early diagnosis or treatment targets [5]. In other cases, TSVs can affect genes by causing a previously non-transcribed region to be incorporated into a gene, causing disruption to the function of the altered gene. There have been fewer studies on these TSVs between transcribed and non-transcribed regions, but their ability to alter downstream RNA and protein structure is likely to lead to similar results as fusion-gene TSVs.

Genomic SVs are typically detected from whole-genome sequencing (WGS) data by identifying reads and read pairs that are incompatible with a reference genome (e.g., [6–11]). However, WGS data are not completely suitable for inferring TSVs since they neither inform which region is transcribed nor reveal how the transcribed sequence will change if SVs alter a splicing site or the stop codon. In addition, WGS data are scarcer and more expensive to obtain than RNA-seq measurements [12], which target transcribed regions directly. RNA-seq is relatively inexpensive, high-throughput, and widely available in many existing and growing data repositories. For example, The Cancer Genome Atlas (TCGA, https://cancergenome.nih.gov) contains RNA-seq measurements from thousands of tumor samples across various cancer types, but 80% of tumor samples in TCGA have RNA-seq data but no WGS data (Additional file 1: Figure S1). While methods exist to detect fusion genes from RNA-seq measurements (e.g., [13–21]), fusion genes are only a subset of TSVs, and existing fusion-gene detection methods rely heavily on current gene annotations and are generally not able or at least not optimized to predict non-fusion-gene TSV events. De novo transcript assembly (e.g., [22–25]) followed by transcript-to-genome alignment (e.g., [26–28]) is used in some fusion-gene detection methods. These approaches rely on annotation-based filtering steps to achieve high accuracy. Although it is possible to extend these approaches to non-fusion-gene TSV detection, the lack of annotation information for non-transcribing regions makes these approaches less suitable for finding non-fusion-gene TSVs. This motivates the need for a method to detect both types of TSVs directly from RNA-seq data.

We present SQUID, the first computational tool that is designed to predict both types of TSVs comprehensively from RNA-seq data. SQUID divides the reference genome into segments and builds a genome segment graph (GSG) from both concordant and discordant RNA-seq read alignments. Using an efficient, novel integer linear program (ILP), SQUID rearranges the segments in the GSG so that as many read alignments as possible are concordant with the rearranged sequence. TSVs are represented by pairs of breakpoints realized by the rearrangement. In this way, it can detect both fusion-gene events and TSVs incorporating previously non-transcribed regions in transcripts. Discordant reads that cannot be made concordant through the optimal rearrangement given by the ILP are discarded as false positive discordant reads, likely due to misalignments. By building a consistent model of the entire rearranged genome and maximizing the number of overall concordant read alignments, SQUID drastically reduces the number of spurious TSVs reported compared with other methods.

SQUID is highly accurate. It is usually >20*%* more accurate than applying WGS-based SV detection methods to RNA-seq data directly. It is similarly more accurate than the pipeline that uses de novo transcript assembly and transcript-to-genome alignment to detect TSVs. We also show that SQUID is able to detect more TSVs involving non-transcribed regions than any existing fusion-gene detection method.

We use SQUID to detect TSVs within 401 TCGA tumor samples of four cancer types (99–101 samples each of breast invasive carcinoma [29], bladder urothelial carcinoma [30], lung adenocarcinoma [31], and prostate adenocarcinoma [32]). SQUID’s predictions suggest that breast invasive carcinoma has a larger variance in terms of number of TSVs/non-fusion-gene TSVs per sample than other cancer types. We also characterize the differences between fusion-gene TSVs and non-fusion-gene TSVs. Observed non-fusion-gene TSVs, for example, are more likely to be intra-chromosomal events. We show that similar breakpoints can occur in multiple samples, and among those that do repeatedly occur, their breakpoint partners are also often conserved. Finally, we identify several novel non-fusion-gene TSVs that affect known tumor suppressor genes (TSGs), which may result in loss of function of corresponding proteins and play a role in tumorigenesis.

## Results

### A novel algorithm for detecting TSVs from RNA-seq

**Fig. 1:**
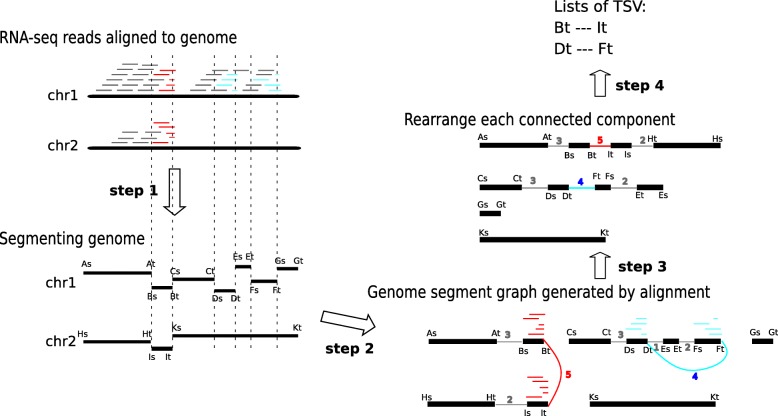
Overview of the SQUID algorithm. Based on the alignments of RNA-seq reads to the reference genome, SQUID partitions the genome into segments (step 1), connects the endpoints of the segments to indicate the actual adjacency in transcript (step 2), and finally reorders the endpoints along the most reliable path (step 3). Thick black lines are genome sequences or segments. Grey, red, and cyan short lines are read alignments, where grey represents concordant alignment, and red and cyan represent discordant alignments of different candidate TSVs. Vertical dashed lines are the separation boundaries between genome segments, and the boundaries are derived based on read alignments. The heads of genome segments are denoted by As, Bs, etc., and the tails are denoted by At, Bt, etc. Step 2: Each read alignment generates one edge between segment endpoints. An edge is added in the following way. When traversing the genome segments along the edge to generate a new sequence, the read can be aligned concordantly onto the new sequence. Multi-edges are collapsed into one weighted edge, where the weight is the number of reads supporting that edge. Red and cyan edges correspond to different candidate TSVs. Step 3: Genome segments are reordered and reoriented to maximize the total number of concordant alignments (concordant edge weights) with respect to the new sequence. Step 4: Discordant edges that are concordant after rearrangement are output as TSVs (in this case, both red edges and cyan edges are output). chr chromosome, TSV transcriptomic structural variant

SQUID predicts TSVs from RNA-seq alignments to the genome (Fig. 1 provides an overview). To do this, it seeks to rearrange the reference genome to make as many of the observed alignments consistent with the rearranged genome as possible. Formally, SQUID constructs a graph from the alignments where the nodes represent boundaries of genome segments and the edges represent adjacencies implied by the alignments. These edges represent both concordant and discordant alignments, where concordant alignments are those consistent with the reference genome and discordant alignments are those that are not. SQUID then uses ILP to order and orient the vertices of the graph to make as many edges consistent as possible. Adjacencies that are present in this rearranged genome but not present in the original reference are proposed as predicted TSVs. The identification of concordant and discordant alignments, construction of the genome segments, creation of the graph, and the reordering objective function are described in the “Methods” section.

### SQUID is accurate on simulated data

**Fig. 2:**
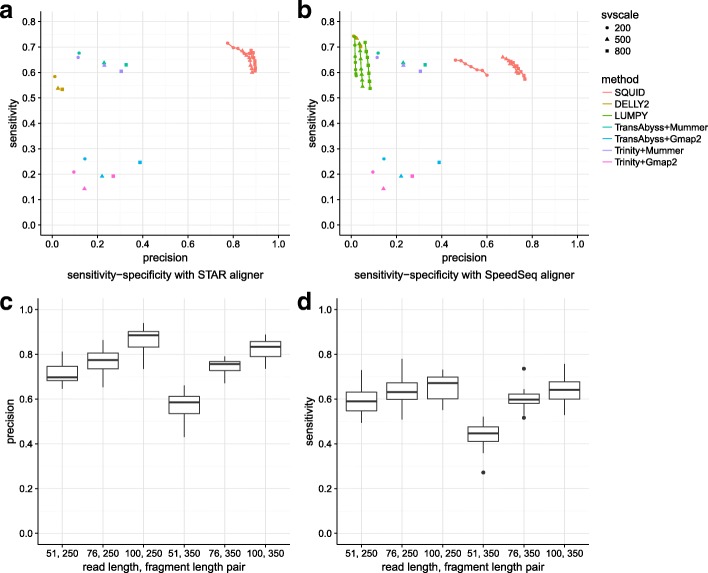
Performance of SQUID and other methods on simulation data. **a**, **b** Different numbers of SVs (200, 500, and 800 SVs) are simulated in each dataset. Each simulated read is aligned with the aligners (**a**) STAR and (**b**) SpeedSeq. If the method allows for user-defined minimum read support for prediction, we vary the threshold from 3 to 9, and plot a sensitivity–precision curve (SQUID and LUMPY), otherwise it is shown as a single point. **c**, **d** Performance of SQUID under different RNA-seq experimental parameter combinations (read lengths of 51, 76, and 100 bp combined with fragment lengths of 250 and 350). A longer read length increases both the precision and sensitivity of SQUID. A longer fragment length slightly decreases SQUID’s performance. A short read length with a long fragment length leads to the worst precision and sensitivity. SV structural variant

Overall, SQUID’s predictions of TSVs are far more precise than other approaches at similar sensitivity on simulated data. SQUID achieves 60% to 80% precision and about 50% sensitivity on simulation data (Fig. 2a, b). SQUID’s precision is >20% higher than several de novo transcriptome assembly and transcript-to-genome alignment pipelines (for details see Additional file 1: Additional Text), and the precision of WGS-based SV detection methods on RNA-seq data is even lower. The sensitivity of SQUID is similar to de novo assembly with MUMmer3 [26], but a little lower than DELLY2 [6] and LUMPY [7] with the SpeedSeq [33] aligner. The overall sensitivity is not as high as the precision, which is probably because there are not enough supporting reads aligned correctly to some TSV breakpoints. That assembly and WGS-based SV detection methods achieve similar sensitivity corroborates the hypothesis that the data limit the achievable sensitivity.

We test SQUID’s robustness to various parameter choices of SQUID itself (Additional file 1: Table S1). SQUID is robust against different values of the segment degree threshold (Additional file 1: Figure S2a, b), which filters edges from segments that are connected to too many other segments. Another parameter, the edge weight threshold, is equivalent to the read support threshold in other SV detection software. It controls the precision–sensitivity tradeoff (Additional file 1: Figure S2c, d). The discordant edge weight coefficient, which up-weights initially discordant reads to compensate for heterogeneous mixtures, does not affect precision or sensitivity in simulation data because simulated reads are homogeneous, and there is no need to adjust for the normal/tumor cell ratio (Additional file 1: Figure S2e, f).

We also test the robustness of SQUID against different RNA-seq experimental settings. Specifically, we simulate RNA-seq data with read lengths of 51, 76 and 100 bp combined with fragment lengths of 250 bp and 350 bp (Fig. 2c, d). For the full table showing the accuracy of SQUID and other methods, see Additional file 2. Each experimental setting has four replicates. With increased read length, SQUID in general performs better in both precision and sensitivity (although there are a few exceptions where the randomness of the simulation shadows the benefit from the longer read length). However, with increased fragment length, SQUID performs slightly worse. In this case, there are fewer reads aligned at the exact breakpoint, possibly due to an increase in split-alignment difficulty for aligners. A short read length (51 bp) with long fragment length (350 bp) leads to the worst precision and sensitivity.

The low precision of the pipeline- and WGS-based methods (Fig. 2) shows that neither of these types of approaches are suitable for TSV detection from RNA-seq data. WGS-based SV detection methods are able to detect TSV signals, but not able to filter out false positives. Assembly-based approaches require solving the transcriptome assembly problem, which is a harder and more time-consuming problem, and thus, errors are more easily introduced. Further, the performance of assembly pipelines depends heavily on the choice of software. For example, MUMmer3 [26] is better at discordantly aligning transcripts than GMAP [27]. Dissect [28] is another transcript-to-genome alignment method that is designed for when SVs exist. (Unfortunately, Dissect did not run to completion on some of the dataset tested here.) It is possible that different combinations of de novo transcript assembly and transcript-to-genome alignment tools can improve the accuracy of the pipelines, but optimizing the pipeline is out of the scope of this work.

SQUID’s effectiveness is likely due to its unified model for both concordant reads and discordant reads. Coverage in RNA-seq alignment is generally proportional to the expression level of the transcript, and using one read count threshold for TSV evidence is not appropriate. Instead, the ILP in SQUID puts concordant and discordant alignments into competition and selects the winner as the most reliable TSV.

### SQUID is able to detect non-fusion-gene TSVs for two previously studied cell lines

Fusion-gene events are a strict subset of TSVs where the two breakpoints are each within a gene region and the fused sequence corresponds to the sense strand of both genes. Fusion genes, thus, exclude TSV events where a gene region is fused with an intergenic region or an anti-sense strand of another gene. Nevertheless, fusion genes have been implicated in playing a role in cancer.

To probe SQUID’s ability to detect both fusion-gene and non-fusion-gene TSVs from real data, we use two cell lines, HCC1954 and HCC1395, both of which are tumor epithelial cells derived from breast. Previous studies have experimentally validated the predicted SVs and fusion-gene events for these two cell lines. Specifically, we compile results from [34–38] for HCC1954 and results from [13, 35] for HCC1395. After removing short deletions and overlapping SVs from different studies, we have 326 validated SVs for the HCC1954 cell line, of which 245 have at least one breakpoint outside a gene region, and the rest (81) have both breakpoints within a gene region. In addition, we have 256 validated true SVs for the HCC1395 cell line, of which 94 have at least one breakpoint outside a gene region, while the rest (162) have both breakpoints within a gene. For a predicted SV to be a true positive, both predicted breakpoints should be within a window of 30 kb of true breakpoints and the predicted orientation should agree with the true orientation. We use a relatively large window, since the true breakpoints can be located within an intron or other non-transcribed region, while the observed breakpoint from RNA-seq reads will be at a nearby coding or expressed region.

We use publicly available RNA-seq data from the Sequencing Read Archive (SRA) of the National Institutes of Health (SRA accessions SRR2532344 [39] and SRR925710 [40] for HCC1954, and SRR2532336 [41] for HCC1395). Because the data are from a pool of experiments, the sample from which RNA-seq was collected may be different from those used for experimental validation. We align reads to the reference genome using STAR. We compare the result with the top fusion-gene detection tools evaluated in [42] and newer software not evaluated by [42], specifically, SOAPfuse [20], deFuse [14], FusionCatcher [16], JAFFA [15], and INTEGRATE [15]. In addition, we compare to the same pipeline of de novo transcriptome assembly and transcript-to-genome alignment as in the previous section (also see Additional file 1: Additional Text). Trans-ABySS [22] is chosen for the de novo transcriptome assembly and MUMmer3 [26] is chosen for the transcript-to-genome alignment, because this combination performed the best with simulation data. Table 1 summarizes the total number of predicted TSVs, and the number of TSVs corresponding to previously validated TSVs (hits). For the full list of TSV predictions by SQUID for the two cell lines, refer to Additional files 3 and 4.

**Table 1:** Summary of TSV predictions for HCC1954 and HCC1395 cell lines

Method		SQUID	FusionCatcher	JAFFA	deFuse	INTEGRATE	SOAPfuse	Pipeline
HCC1954	Fusion-gene predictions	46	54	67	95	67	177	2118
	Fusion-gene hits	7	5	4	12	10	5	4
	Non-fusion-gene predictions	46	0	0	83	0	0	1080
	Non-fusion-gene hits	7	0	0	5	0	0	11
HCC1395	Fusion-gene predictions	44	42	44	110	61	185	2413
	Fusion-gene hits	11	11	16	15	16	19	23
	Non-fusion-gene predictions	57	0	0	121	0	0	1185
	Non-fusion-gene hits	9	0	0	7	0	0	8

**Fig. 3:**
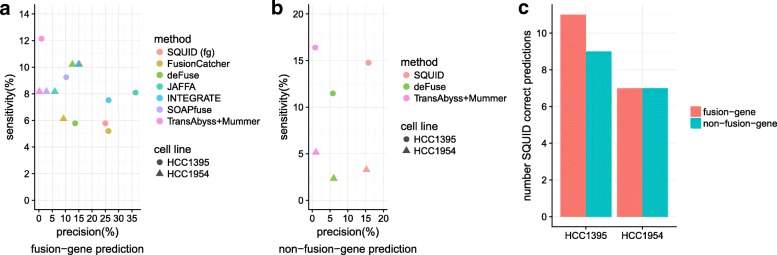
Performance of SQUID and fusion-gene detection methods on breast cancer cell lines HCC1954 and HCC1395. Predictions are evaluated for previously validated SVs and fusions. **a** Fusion-gene prediction sensitivity–precision curve of different methods. **b** Non-fusion-gene prediction sensitivity–precision curve. Only SQUID, deFuse, and the pipeline of de novo transcriptome assembly and transcript-to-genome alignment are able to predict non-fusion-gene TSVs. **c** Number of fusion-gene TSVs and non-fusion-gene TSVs correctly predicted by SQUID. Non-fusion-gene TSVs make up a considerable proportion of all TSVs. TSV transcriptomic structural variant

When restricted to fusion-gene events, SQUID achieves similar precision and sensitivity compared to fusion-gene detection tools (Fig. 3a). These methods have different rankings for the two cell lines. There is no uniformly best method for fusion-gene TSV predictions for both cell lines. SQUID has one of the highest precision values and the second highest sensitivity for the HCC1954 cell line and ranks in the middle for the HCC1395 cell line. For both cell lines, the pipeline of de novo transcriptome assembly and transcript-to-genome alignment has very low precision, which suggests that without filtering steps, assembly-based methods are not able to distinguish between noise and true TSVs.

It is even harder to predict non-fusion-gene TSVs accurately, since current annotations cannot be used to limit the search space for potential read alignments or TSV events. Only SQUID, deFuse, and the pipeline of de novo transcriptome assembly and transcript-to-genome alignment are able to detect non-fusion-gene events. SQUID has both a higher precision and a higher sensitivity compared to deFuse (Fig. 3b). The assembly pipeline has a higher sensitivity but very low precision, which again indicates that this pipeline outputs non-fusion-gene TSV signals without distinguishing them from noise. A considerable proportion of validated TSVs are non-fusion-gene TSVs. Correctly predicted non-fusion-gene TSVs make up almost half of all correct predictions of SQUID (Fig. 3c).

We test the robustness of SQUID with respect to different parameter values on the two cell lines (Additional file 3: Figure S3). We find the same trend regarding the segment degree threshold and the edge weight threshold as with simulated data. The segment degree threshold does not affect either precision or sensitivity much, and the edge weight threshold determines the precision–sensitivity tradeoff. The discordant edge weight coefficient does not change the sensitivity on the HCC1954 cell line, possibly indicating that the sequencing data is relatively homogeneous. As this parameter increases, precision for the HCC1954 cell line slightly decreases because more TSVs are predicted. In contrast, an increase of the discordant edge weight coefficient increases both the precision and sensitivity of the HCC1395 cell line. This implies that for some transcripts, normal reads dominate tumor reads, and increasing this parameter allows us to identify those TSVs.

The sensitivity for both cell lines of all tested methods is relatively low. One explanation for this is the difference between the source of the data used for prediction and validation. In the ground truth, some SVs were first identified using WGS data or BAC end sequencing and then validated experimentally. Not all genes are expressed in the RNA-seq data used here, and lowly expressed genes may not generate reads spanning SV breakpoints due to read sampling randomness. To quantify the feasibility of each SV being detected, we count the number of supporting chimeric reads in RNA-seq alignments. The proportion of ground-truth fusion-gene TSVs with supporting reads is very low for both cell lines: 26.5% for HCC1954 (13 out of 49) and 27.1% for HCC1395 (47 out of 173). The maximum sensitivity of fusion-gene TSV prediction is limited by these numbers, which explains the relatively low sensitivity we observed. For non-fusion-gene TSVs, only 13.0% in HCC1954 (36 out of 277) and 21.7% in HCC1395 (13 out of 83) can possibly be identified.

We use WGS data for the corresponding cell lines to validate the novel TSVs predicted by SQUID (SRA accession numbers ERP000265 [43] for HCC1954 and SRR892417 [44] and SRR892296 [45] for HCC1395). For each TSV prediction, we extract a 25-kb sequence around both breakpoints and concatenate them according to the predicted TSV orientation. We then map the WGS reads against these junction sequences using SpeedSeq [33]. If a paired-end WGS read can be mapped only concordantly to a junction sequence but not to the reference genome, that paired-end read is marked as supporting the TSV. If at least three WGS reads support a TSV, the TSV is considered as validated. With this approach, we are able to validate 40 more TSV predictions for the HCC1395 cell line and 18 more TSV predictions for the HCC1954 cell line. In total, the percentage of predicted TSVs that can be validated either by previous studies or by WGS data is 57.7% for the HCC1395 cell line and 35.2% for the HCC1954 cell line. The WGS validation rate of the HCC1954 cell line is much lower than for the HCC1395 cell line, which can be explained by the relatively low read depth. The read depth for HCC1954 WGS data is 7.6 × and that for HCC1395 WGS data is 22.7 ×.

### Characterizing TSVs on four types of TCGA cancer samples

To compare the distributions and characteristics of TSVs among cancer types and between TSV types, we applied SQUID on arbitrarily selected 99 to 101 tumor samples from TCGA for each of four cancer types: breast invasive carcinoma (BRCA), bladder urothelial carcinoma (BLCA), lung adenocarcinoma (LUAD), and prostate adenocarcinoma (PRAD). TCGA aliquot barcodes of the corresponding samples are listed in Additional file 5. For data processing details, see Additional file 1: Additional Text. The running time of SQUID is less than 3 hours for the majority of the RNA-seq data we selected, and the maximum memory usage is around 4 or 8 GB (Additional file 1: Figure S4).

To estimate the accuracy of SQUID’s prediction for selected TCGA samples, we use WGS data for the same patients to validate TSV junctions. There are 72 WGS experiments available for the 400 samples (20 BLCA, 10 BRCA, 31 LUAD, and 11 PRAD). We use the same approach with WGS to validate SQUID predictions as in the previous section. SQUID’s overall validation rate is 88.21%, which indicates that SQUID is quite accurate and reliable on TCGA data.

**Fig. 4:**
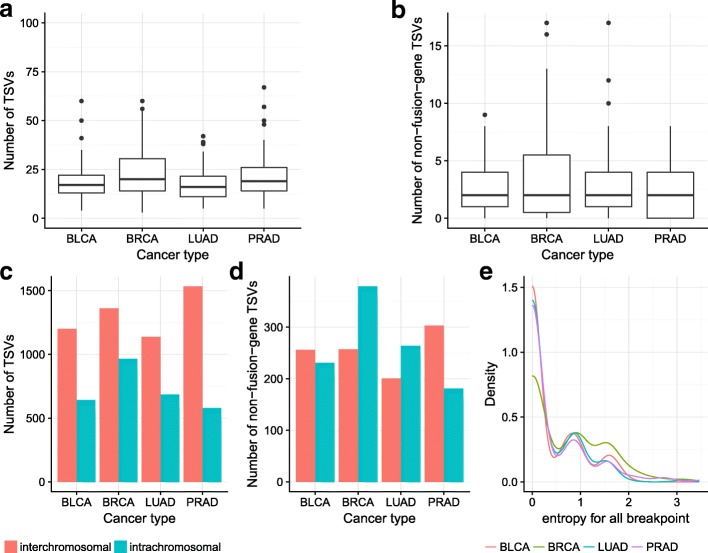
**a**, **b** Number of TSVs and non-fusion-gene TSVs in each sample in different cancer types. BRCA has slightly more samples with a larger number of (non-fusion-gene) TSVs. Thus, it has a longer tail on the *y*-axis. **c**, **d** Number of inter-chromosomal and intra-chromosomal TSVs within all TSVs and within non-fusion-gene TSVs. Non-fusion-gene TSVs contain more intra-chromosomal events than fusion-gene TSVs. (**e**) For breakpoints occurring more than 3 times in the same cancer type, the distribution of the entropy of its TSV partner. The lower the entropy, the more likely it is that the breakpoint has a fixed partner. The peak near 0 indicates a large portion of breakpoints are likely to be rejoined with the same partner in the TSV. However, there are still some breakpoints that have multiple rejoined partners. BLCA bladder urothelial carcinoma, BRCA breast invasive carcinoma, LUAD lung adenocarcinoma, PRAD prostate adenocarcinoma, TSV transcriptomic structural variant

We find that most samples have ∼18–23 TSVs, including ∼3–4 non-fusion-gene TSVs among all four cancer types (Fig. 4a, b). For BRCA, the tail of the distribution of TSV counts is larger, and more samples contain a larger number of TSVs. The same trend is observed when restricted to non-fusion-gene TSVs.

Inter-chromosomal TSVs are more prevalent than intra-chromosomal TSVs for all cancer types (Fig. 4c), although this difference is much more pronounced in bladder and prostate cancer. Non-fusion-gene TSVs are more likely to be intra-chromosomal events than fusion-gene TSVs (Fig. 4d), and in fact in breast and lung cancer, we detect more intra-chromosomal non-fusion-gene TSVs than inter-chromosomal non-fusion-gene TSVs. Prostate cancer is an exception in that, for non-fusion-gene TSVs, inter-chromosomal events are observed much more often than intra-chromosomal events. Nevertheless, non-fusion-gene TSVs are more likely to be intra-chromosomal than fusion-gene TSVs, because the percentage of intra-chromosomal TSVs within non-fusion-gene TSVs is higher than that within all TSVs.

For a large proportion of breakpoints occurring multiple times within a cancer type, their partner in the TSV is likely to be fixed and to reoccur every time that breakpoint is used. To quantify this, for each breakpoint that occurred ≥3 times, we compute the entropy of its partner promiscuity. Specifically, we derive a discrete empirical probability distribution of partners for each breakpoint and compute the entropy of this distribution. This measure, thus, represents the uncertainty of the partner given one breakpoint, with higher entropy corresponding to a less conserved partnering pattern. In Fig. 4e, we see that there is a high peak near 0 for all cancer types, which indicates that for a large proportion of recurring breakpoints, we are certain about its rejoined partner once we know the breakpoint. However, there are also promiscuous breakpoints with entropy larger than 0.5.

### Tumor suppressor genes can undergo TSV and generate altered transcripts

TSGs protect cells from becoming cancer cells. Usually their functions involve inhibiting cell cycle, facilitating apoptosis, and so on [46]. Mutations in TSGs may lead to loss of function of the corresponding proteins and benefit tumor growth. For example, a homozygous loss-of-function mutation in p53 is found in about half of cancer samples across various cancer types [47]. TSVs are likely to cause loss of function of TSGs as well. Indeed, we observe several TSGs that are affected by TSVs, both of the fusion-gene type and the non-fusion-gene type.

**Fig. 5:**
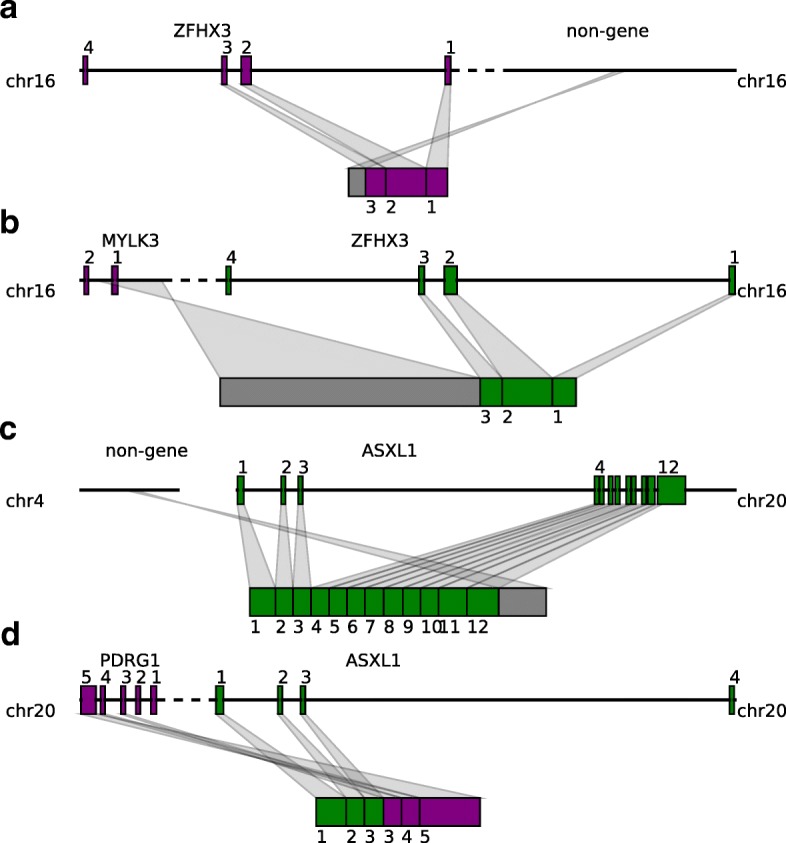
Tumor suppressor genes are affected by both fusion-gene and non-fusion-gene TSVs and generate transcripts with various features. **a** *ZFHX3* is fused with an intergenic region after exon 3. The transcript stops at the inserted region, losing the rest of the exons. **b** *ZFHX3* is fused with a part of the *MYLK3* anti-sense strand after exon 3. Codon and splicing signals are not preserved on anti-sense strands, thus, an *MYLK3* anti-sense insertion acts like an intergenic region insertion and causes transcription to stop before reaching the rest of the *ZFHX3* exons. **c** *ASXL1* is fused with an intergenic region in the middle of exon 12. The resulting transcript contains a truncated *ASXL1* exon 12 and intergenic sequence. **d** The first three exons of the *ASXL1* gene are joined with the last three exons of *PDRG1*, resulting in a fused transcript containing six complete exons from both *ASXL1* and *PDRG1*. chr chromosome, TSV transcriptomic structural variant

The *ZFHX3* gene encodes a transcription factor that transactivates cyclin-dependent kinase inhibitor 1A (*CDKN1A*), a cell cycle inhibitor [48]. We find that in one BLCA and one BRCA sample, there are TSVs affecting *ZFHX3*. These two TSVs events are different from each other in terms of the breakpoint partner outside of *ZFHX3*. In the BLCA tumor sample, an intergenic region is inserted after the third exon of *ZFHX3* (see Fig. 5a; for a visualization with Integrative Genomics Viewer (IGV) [49], see Additional file 1: Figure S5). The fused transcript stops at the inserted region, causing the *ZFHX3* transcript to lose the rest of its exons. In the BRCA tumor sample, a region of the anti-sense strand of gene *MYLK3* is inserted after the third exon of the *ZFHX3* gene (Fig. 5b, Additional file 1: Figure S6). Because codons and splicing sites are not preserved on the anti-sense strand, the transcribed insertion region does not correspond to known exons of the *MYLK3* gene, but covers the range of the first exon of *MYLK3* and extends to the first intron and 5^′^ intergenic region. Transcription stops within the inserted region, and causes the *ZFHX3* transcript to lose exons after exon 3, which resembles the fusion with the intergenic region in the BLCA sample.

Another example is given by the *ASXL1* gene, which is essential for activating *CDKN2B* to inhibit tumorigenesis [50]. We observe two distinct TSVs related to *ASXL1* from BLCA and BRCA samples. The first TSV merges the first 11 exons and half of exon 12 of *ASXL1* with an intergenic region on chromosome 4 (Fig. 5c, Additional file 1: Figure S7). Transcription stops at the inserted intergenic region, leaving the rest of exon 12 untranscribed. The breakpoint within *ASXL1* is before the 3^′^ untranslated region, so the downstream protein sequence from exon 12 will be affected. The other TSV involving *ASXL1* is a typical fusion-gene TSV where the first three exons of *ASXL1* are fused with the last three exons from the *PDRG1* gene (Fig. 5d, Additional file 1: Figure S8). Protein domains after *ASXL1* exon 4 and before *PDRG1* exon 2 are lost in the fused transcript.

These non-fusion-gene examples are novel predicted TSV events, which are not typically detectable via traditional fusion-gene detection methods using RNA-seq data. They suggest that non-fusion-gene events can also be involved in tumorigenesis by disrupting TSGs.

## Discussion

SQUID is able to predict both traditional fusion-gene TSVs and non-fusion-gene TSVs from RNA-seq data with high accuracy. This is due to its unique approach to predicting TSVs, whereby it constructs a consistent model of the underlying rearranged genome that explains as much of the data as possible. In particular, it simultaneously considers both concordant and discordant reads, and by rearranging genome segments to maximize the number of concordant reads, SQUID generates a set of compatible TSVs that are most reliable in terms of the numbers of reads supporting them. Instead of a universal read support threshold, the objective function in SQUID naturally balances reads supporting and not supporting a candidate TSV. This design is efficient in filtering out sequencing and alignment noise in RNA-seq, especially in the annotation-free context of predicting non-fusion-gene TSV events.

By applying SQUID to TCGA RNA-seq data, we are able to detect TSVs in cancer samples, especially non-fusion-gene TSVs. We identify novel non-fusion-gene TSVs involving the known TSGs *ZFHX3* and *ASXL1*. Both fusion-gene and non-fusion-gene events detected in TCGA samples are computational predictions and need further experimental validation.

Other important uses and implications for general TSVs have yet to be explored and represent possible directions for future work. TSVs will impact the accuracy of transcriptome assembly and expression quantification, and methodological advances are needed to correct those downstream analyses for the effect of TSVs. For example, current reference-based transcriptome assemblers are not able to assemble from different chromosomes for inter-chromosomal TSVs. In addition, expression levels of TSV-affected transcripts cannot be quantified if they are not present in the transcript database. Incorporating TSVs into transcriptome assembly and expression quantification can potentially improve their accuracy. SQUID’s ability to provide a new genome sequence that is as consistent as possible with the observed reads will facilitate its use as a preprocessing step for transcriptome assembly and expression quantification, though optimizing this pipeline remains a task for future work.

Several natural directions exist for extending SQUID. First, SQUID is not able to predict small deletions. Instead, it treats small deletions the same as introns. This is to some extent a limitation of using RNA-seq data. Introns and deletions are difficult to distinguish, as both result in concordant split reads or stretched mate pairs. Using gene annotations could somewhat address this problem. Second, when the RNA-seq reads are derived from a highly heterogeneous sample, SQUID is likely not able to predict all TSVs in the same region if they conflict, since it seeks a single consistent genome model. Instead, SQUID will pick only the dominating one that is compatible with other predicted TSVs. One approach to handle this would be to re-run SQUID iteratively, removing reads that are explained at each step. Again, this represents an attractive avenue for future work.

SQUID is open source and available at http://www.github.com/Kingsford-Group/squid and the scripts to replicate the computational experiments described here are available at http://www.github.com/Kingsford-Group/squidtest.

## Conclusion

We developed SQUID, the first algorithm to detect TSVs accurately and comprehensively that targets both traditional fusion-gene detection and the much broader class of general TSVs. SQUID exhibits higher precision at similar sensitivities compared with WGS-based SV detection methods and pipelines of de novo transcriptome assembly and transcript-to-genome alignment. In addition, it can detect non-fusion-gene TSVs with similarly high accuracy.

We use SQUID to predict TSVs in TCGA tumor samples. From our prediction, BRCA has a slightly flatter distribution of the number of per-sample TSVs than the other cancer types studied. We observe that non-fusion-gene TSVs are more likely to be intra-chromosomal events than fusion-gene TSVs. This is likely due to the different sequence composition features in gene vs. non-gene regions. PRAD also stands out because it has the largest percentage of inter-chromosomal TSVs. Overall, these findings continue to suggest that different cancer types have different preferred patterns of TSVs, although the question remains whether these differences will hold up as more samples are analyzed and whether the different patterns are causal, correlated, or mostly due to non-functional randomness. These findings await experimental validation.

As shown by predictions from SQUID, TSGs are involved in non-fusion-gene TSVs. In these cases, transcription usually stops within the inserted region of the non-fusion-gene TSVs, which causes the TSG transcript to lose some of its exons, and possibly leads to downstream loss of function. The large-scale variations of TSGs suggest that non-fusion-gene TSVs can potentially affect cancer genesis and progression, and needs to be studied more carefully.

## Methods

### The computational problem: rearrangement of genome segments

**Fig. 6:**
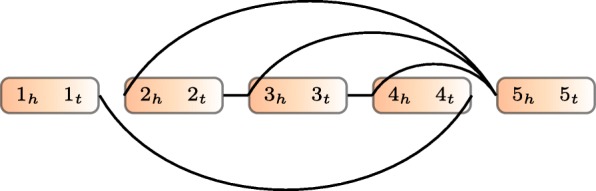
Example of a genome segment graph. Boxes are genome segments, each of which has two ends with subscripts *h* and *t*. The color gradient indicates the orientation from head to tail. Edges connect the ends of genome segments

We formulate the TSV detection problem as the optimization problem of rearranging genome segments to maximize the number of observed reads that are consistent (termed *concordant*) with the rearranged genome. This approach requires defining the genome segments that can be independently rearranged. It also requires defining which reads are consistent with a particular arrangement of the segments. We will encode both of these (segments and read consistency) within a GSG. Fig. 6 is an example.

#### **Definition 1**

(Segment) A *segment* is a pair *s*=(*s*_*h*_,*s*_*t*_), where *s* represents a continuous sequence in the reference genome. *s*_*h*_ represents its head and *s*_*t*_ its tail in reference genome coordinates. In practice, segments will be derived from read locations.

#### **Definition 2**

(Genome Segment Graph (GSG)) A GSG *G*=(*V*,*E*,*w*) is an undirected weighted graph, where *V* contains both endpoints of each segment in a set of segments *S*, i.e., *V*={*s*_*h*_:*s*∈*S*}∪{*s*_*t*_:*s*∈*S*}. Thus, each vertex in the GSG represents a location in the genome. An edge (*u*,*v*)∈*E* indicates that there is evidence that the location *u* is adjacent to location *v*. The weight function $w: E \longrightarrow \mathbb {R}^{+}$ represents the reliability of an edge. Generally speaking, the weight is the number of read alignments supporting the edge, but we use a multiplier to calculate edge weights, which is discussed below. In practice, *E* and *w* will be derived from split-aligned and paired-end reads.

Defining vertices by endpoints of segments is required to avoid ambiguity. Knowing only that segment *i* is connected with segment *j* is not enough to recover the sequence, since different relative positions of *i* and *j* spell out different sequences. Instead, for example, an edge (*i*_*t*_,*j*_*h*_) indicates that the tail of segment *i* is connected to the head of segment *j*, and this specifies a unique desired local sequence with the only other possibility being the reverse complement (i.e., it could be that the true sequence is *i*·*j* or rev(*j*)· rev(*i*); here · indicates concatenation and rev(*i*) is the reverse complement of segment *i*).

A GSG is similar to a breakpoint graph [51] but with critical differences. A breakpoint graph has edges representing connections both in the reference genome and in the target genome. Edges in a GSG represent only the target genome, and they can be either concordant or discordant. In addition, a GSG does not require that the degree of every vertex is 2, and thus, alternative splicing and erroneous edges can exist in a GSG.

Our goal is to reorder and reorient the segments in *S* so that as many edges in *G* are compatible with the rearranged genome as possible.

#### **Definition 3**

(Permutation) A permutation *π* on a set of segments *S* projects a segment in *S* to a set of integers from 1 to |*S*| (the size of *S*), representing the indices of the segments in an ordering of *S*. In other words, each permutation *π* defines a new order of segments in *S*.

#### **Definition 4**

(Orientation Function) An orientation function *f* maps both ends of a segment to 0 or 1:

$$ f:\{s_{h}: s\in S\}\cup\{s_{t}: s\in S\} \longrightarrow \{0,1\}, $$

subject to *f*(*s*_*h*_)+*f*(*s*_*t*_)=1 for all *s*=(*s*_*h*_,*s*_*t*_)∈*S*. An orientation function specifies the orientations of all segments in *S*. Specifically, *f*(*s*_*h*_)=1 means *s*_*h*_ goes first and *s*_*t*_ next, corresponding to the forward strand of the segment, and *f*(*s*_*t*_)=1 corresponds to the reverse strand of the segment.

With a permutation *π* and an orientation function *f*, the exact and unique sequence of a genome is determined. The reference genome also corresponds to a permutation and an orientation function, where the permutation is the identity permutation and the orientation function maps all *s*_*h*_ to 1 and all *s*_*t*_ to 0.

#### **Definition 5**

(Edge Compatibility) Given a set of segments *S*, a GSG *G*=(*V*,*E*,*w*), a permutation *π* on *S*, and an orientation function *f*, an edge *e*=(*u*_*i*_,*v*_*j*_)∈*E*, where *u*_*i*_∈{*u*_*h*_,*u*_*t*_} and *v*_*j*_∈{*v*_*h*_,*v*_*t*_}, is *compatible* with permutation *π* and orientation *f* if and only if

1 $$ 1-f(v_{j})=\mathbf{1}\left[\pi(v)<\pi(u)\right]=f(u_{i}),  $$

where **1**[*x*] is an indicator function, which is 1 if *x* is true and 0 otherwise. Comparison between permuted elements is defined as comparing their index in permutation, that is, *π*(*v*)<*π*(*u*) states that segment *v* is in front of segment *u* in rearrangement *π*. We write *e*∼(*π*,*f*) if *e* is compatible with *π* and *f*.

The above two edge compatibility Eqs. 1 require that, for an edge to be compatible with the rearranged and reoriented sequence determined by *π* and *f*, it needs to connect the right side of the segment in front to the left side of the segment following it. As we will see below, the edges of a GSG are derived from read alignments. An edge being compatible with *π* and *f* is essentially equivalent to stating that the corresponding read alignments are concordant with respect to the target genome determined by *π* and *f*. When (*π*,*f*) is clear, we refer to edges that are compatible as concordant edges and edges that are incompatible as discordant edges.

With the above definitions, we formulate an optimization problem as follows:

#### **Problem 1**

**Input:** A set of segments *S* and a GSG *G*=(*V*,*E*,*w*).**Output:** Permutation *π* on *S* and orientation function *f* that maximizes:

2 $$  \max_{\pi, f} \sum_{e\in E} w(e) \cdot \mathbf{1}\left[e\sim(\pi,f)\right].  $$

This objective function tries to find a rearrangement of genome segments (*π*,*f*) such that when aligning reads to the rearranged sequence, as many reads as possible will be aligned concordantly. This objective function includes both concordant alignments and discordant alignments and sets them in competition, which is effective in reducing the number of false positives when tumor transcripts outnumber normal transcripts. There is the possibility that some rearranged tumor transcripts will be outnumbered by normal counterparts. To be able to detect TSVs in this case, depending on the setting, we may weight discordant read alignments more than concordant read alignments. Specifically, for each discordant edge *e*, we multiply the weight *w*(*e*) by a constant *α* that represents our estimate of the ratio of normal transcripts over tumor counterparts.

The final TSVs are modeled as pairs of breakpoints. Denote the permutation and orientation corresponding to an optimally rearranged genome as (*π*^∗^,*f*^∗^) and those that correspond to the reference genome as (*π*_0_,*f*_0_). An edge *e* can be predicted as a TSV if *e*∼(*π*^∗^,*f*^∗^) and $e\nsim (\pi _{0},f_{0})$.

### Integer linear programming formulation

We use ILP to compute an optimal solution (*π*^∗^,*f*^∗^) of Problem 1. To do this, we introduce the following Boolean variables:

- *x*_*e*_=1 if edge *e*∼(*π*^∗^,*f*^∗^) and 0 if not.
- *z*_*uv*_=1 if segment *u* is before *v* in the permutation *π*^∗^ and 0 otherwise.
- *y*_*u*_=1 if *f*^∗^(*u*_*h*_)=1 for segment *u*.

With this representation, the objective function can be rewritten as

3 $$ \max_{x_{e}, y_{u}, z_{uv}} w(e) \cdot x_{e}.  $$

We add constraints to the ILP derived from edge compatibility Eq. 1. Without loss of generality, we first suppose segment *u* is in front of *v* in the reference genome, and edge *e* connects *u*_*t*_ and *v*_*h*_ (which is a tail–head connection). Plugging in *u*_*t*_, the first equation in (1) is equivalent to 1−**1**[*π*(*u*)>*π*(*v*)]=1−*f*(*u*_*t*_) and can be rewritten as **1**[*π*(*u*)<*π*(*v*)]=*f*(*u*_*h*_)=*y*_*u*_. Note that **1**[*π*(*u*)<*π*(*v*)] has the same meaning as *z*_*uv*_; it leads to the constraint *z*_*uv*_=*y*_*u*_. Similarly, the second equation in (1) indicates *z*_*uv*_=*y*_*v*_. Therefore, *x*_*e*_ can reach 1 only when *y*_*u*_=*y*_*v*_=*z*_*uv*_. This is equivalent to the inequalities (4) below. Analogously, we can write constraints for the other three types of edge connections: tail–tail connections impose inequalities (5), head–head connections impose inequalities (6), and head–tail connections impose inequalities (7):

4 $$  \begin{aligned} x_{e} &\leq y_{u}-y_{v}+1, \\ x_{e} &\leq y_{v}-y_{u}+1, \\ x_{e} &\leq y_{u}-z_{uv}+1, \\ x_{e} &\leq z_{uv}-y_{u}+1, \end{aligned}  $$

5 $$  \begin{aligned} x_{e} &\leq y_{u}-(1-y_{v})+1, \\ x_{e} &\leq (1-y_{v})-y_{u}+1, \\ x_{e} &\leq y_{u}-z_{uv}+1, \\ x_{e} &\leq z_{uv}-y_{u}+1, \end{aligned}  $$

6 $$  \begin{aligned} x_{e} &\leq (1-y_{u})-y_{v}+1, \\ x_{e} &\leq y_{v}-(1-y_{u})+1, \\ x_{e} &\leq (1-y_{u})-z_{uv}+1, \\ x_{e} &\leq z_{uv}-(1-y_{u})+1, \end{aligned}  $$

7 $$  \begin{aligned} x_{e} &\leq (1-y_{u})-(1-y_{v})+1, \\ x_{e} &\leq (1-y_{v})-(1-y_{u})+1, \\ x_{e} &\leq (1-y_{u})-z_{uv}+1, \\ x_{e} &\leq z_{uv}-(1-y_{u})+1. \end{aligned}  $$

We also add constraints to enforce that *z*_*uv*_ forms a valid topological ordering. For each pair of nodes *u* and *v*, one must be in front of the other, that is *z*_*uv*_+*z*_*vu*_=1. In addition, for each triple of nodes, *u*, *v*, and *w*, one must be at the beginning and one must be at the end. Therefore, we add 1≤*z*_*uv*_+*z*_*vw*_+*z*_*wu*_≤2.

Solving an ILP in theory takes exponential time, but in practice, solving the above ILP to rearrange genome segments is very efficient. The key is that we can solve for each connected component separately. Because the objective maximizes the sum of compatible edge weights, the best rearrangement of one connected component is independent of the rearrangement of another because, by definition, there are no edges between connected components.

### Concordant and discordant alignments

**Fig. 7:**
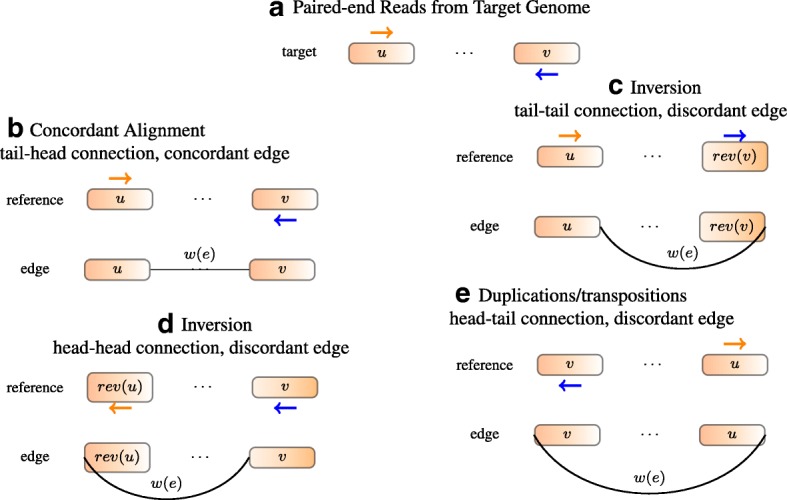
Constructing edges from alignment. **a** Read positions and orientations generated from the target genome. **b** If the reference genome does not have rearrangements, the read should be concordantly aligned to the reference genome. An edge is added to connect the right end of *u* to the left end of *v*. Traversing the two segments along the edge reads out *u*·*v*, which is the same as the reference. **c** Both ends of the read align to the forward strand. An edge is added to connect the right end of *u* to the right end of rev(*v*). Traversing the segments along the edge reads out sequence *u*· rev(rev(*v*))=*u*·*v*, which recovers the target sequence and the read can be concordantly aligned. **d** If both ends align to the reverse strand, an edge is added to connect the left end of the front segment to the left end of the back segment. **e** If two ends of a read point away from each other, an edge is added to connect the left end of the front segment to the right end of the back segment

Discordant alignments are alignments of reads that contradict the library preparation in sequencing. Concordant alignments are alignments of reads that agree with the library preparation. Take Illumina sequencing as an example. For a paired-end read alignment to be concordant, one end should be aligned to the forward strand and the other to the reverse strand, and the forward strand aligning position should be in front of the reverse strand aligning position (Fig. 7a). Concordant alignment traditionally used in WGS also requires that a read cannot be split and aligned to different locations. However, these requirements are invalid in RNA-seq alignments because alignments of reads can be separated by an intron with unknown length.

We define concordance criteria separately for split-alignment and paired-end alignment. If one end of a paired-end read is split into several parts and each part is aligned to a location, the end has split alignments. Denote the vector of the split alignments of an end as *R*=[*A*_1_,*A*_2_,…,*A*_*r*_] (*r* depends on the number of splits). Each alignment *R*[*i*]=*A*_*i*_ has four components: a chromosome (Chr), an alignment starting position (Spos), an alignment ending position, and an orientation (Ori, with value either + or −). We require that the alignments *A*_*i*_ are sorted by their position in the read. A split-aligned end *R*=[*A*_1_,*A*_2_,…,*A*_*r*_] is concordant if all the following conditions hold:

8 $$ \begin{aligned} A_{i}.\text{Chr} &= A_{j}.\text{Chr}, && \text{\(\forall i, \forall j\)}, \\ A_{i}.\text{Ori} &= A_{j}.\text{Ori}, && \text{\(\forall i, \forall j\)}, \\ A_{i}.\text{Spos} &< A_{j}.\text{Spos}, && \text{if \(A_{i}.\text{Ori}=+\) for all \(i<j\)}, \\ A_{i}.\text{Spos} &>A_{j}.\text{Spos}, && \text{if \(A_{i}.\text{Ori}=-\) for all \(i<j\)}. \end{aligned}  $$

If the end is not split, but continuously aligned, the alignment automatically satisfies Eq. 8. Denote the alignments of *R*’s mate as *M*=[*B*_1_,*B*_2_,…,*B*_*m*_]. An alignment of the paired-end read is concordant if the following conditions all hold:

9 $$ \begin{aligned} A_{i}.\text{Chr} &= B_{j}.\text{Chr}, && \text{\(\forall i, \forall j\)}, \\ A_{i}.\text{Ori} &\neq B_{j}.\text{Ori}, && \text{\(\forall i, \forall j\)}, \\ A_{1}.\text{Spos} &< B_{m}.\text{Spos}, && \text{if \(A_{1}.\text{Ori}=+\)}, \\ A_{m}.\text{Spos} &> B_{1}.\text{Spos}, && \text{if \(A_{1}.\text{Ori}=-\)}. \end{aligned}  $$

We require only that the leftmost split of the forward read *R* is in front of the leftmost split of the reverse read *M*, since the two ends in a read pair may overlap. For a paired-end read to be concordant, each end should satisfy split-read alignment concordance (8), and the pair should satisfy paired-end alignment concordance (9).

### Splitting the genome into segments *S*

We use a set of breakpoints to partition the genome. The set of breakpoints contains two types of positions: (1) the start position and end position of each interval of overlapping discordant alignments and (2) an arbitrary position in each 0-coverage region.

Ideally, both ends of a discordant read should be in separate segments, otherwise, a discordant read in a single segment will always be discordant, no matter how the segments are rearranged. Assuming discordant read alignments of each TSV pile up around the breakpoints and do not overlap with the discordant alignments of other TSVs, we set a breakpoint on the start and end positions of each contiguous interval of overlapping discordant alignments.

Each segment that contains discordant read alignments may also contain concordant alignments that connect the segment to its adjacent segments. To avoid having all segments in a GSG connected to their adjacent segments and thus, creating one big connected component, we pick the starting point of each 0-coverage region as a breakpoint. By adding those breakpoints, different genes will be in separate connected components unless some discordant reads support their connection. Overall, the size of each connected component is not very large. The number of nodes generated by each gene is approximately the number of exons located in them and these gene subgraphs are connected only when there is a potential TSV between them.

### Defining edges and filtering out obvious false positives

In a GSG, an edge is added between two vertices when there are reads supporting the connection. For each read spanning different segments, we build an edge such that when traversing the segments along the edge, the read is concordant with the new sequence [Eqs. (8) and (9)]. Examples of deriving an edge from a read alignment are given in Fig. 7. In this way, the concordance of an alignment and the compatibility of an edge with respect to a genome sequence are equivalent.

The weight of a concordant edge is the number of read alignments supporting the connection, while the weight of a discordant edge is the number of supporting alignments multiplied by the discordant edge weight coefficient *α*. The discordant edge weight coefficient *α* represents the normal/tumor cell ratio (for a complete table of SQUID parameters, see Additional file 1: Table S1). If normal transcripts dominate tumor transcripts, *α* enlarges the discordant edge weights and helps to satisfy the discordant edges in the rearrangement of the ILP.

We filter out obvious false positive edges to reduce both the ILP computation time and the mistakes after the ILP. Edges with very low read support are likely to be a result of alignment error, therefore, we filter out edges with a weight lower than a threshold *θ*. Segments with too many connections to other regions are likely to have low mappability, so we also filter out segments connecting to more than *γ* other segments. The parameters *α*, *θ*, and *γ* are the most important user-defined parameters in SQUID (Additional file 1: Table S1, Figures S2 and S3). An interleaving structure of exons from different regions (different genes) seems more likely to be a result of sequencing or alignment error rather than an SV. Thus, we filter out the interleaving edges between two such groups of segments.

### Identifying TSV breakpoint locations

Edges that are discordant in the reference genome indicate potential rearrangements in transcripts. Among those edges, some are compatible with the permutation and orientation from ILP. These edges are taken to be the predicted TSVs. For each edge that is discordant initially but compatible with the optimal rearrangement found by ILP, we examine the discordant read alignments to determine the exact breakpoint within related segments. Specifically, for each end of a discordant alignment, if there are two other read alignments that start or end in the same position and support the same edge, then the end of the discordant alignment is predicted to be the exact TSV breakpoint. Otherwise, the boundary of the corresponding segment will be output as the exact TSV breakpoint.

### Simulation methodology

Simulations with randomly added SVs and simulated RNA-seq reads were used to evaluate SQUID’s performance in situations with a known correct answer. RSVsim [52] was used to simulate SVs in the human genome (Ensembl 87 or hg38) [53]. We use the five longest chromosomes for simulation (chromosome 1 to chromosome 5). RSVsim introduces five different types of SVs: deletion, inversion, insertion, duplication, and inter-chromosomal translocation. To vary the complexity of the resulting inference problem, we simulated genomes with 200 SVs of each type, 500 SVs of each type, and 800 SVs of each type. We generated four replicates for each level of SV complexity (200, 500, and 800). For inter-chromosomal translocations, we simulate only two events because only five chromosomes were used.

In the simulated genome with SVs, the original gene annotations are not applicable, and we cannot simulate gene expression from the rearranged genome. Therefore, for testing, we interchange the roles of the reference (hg38) and the rearranged genome, and use the new genome as the reference genome for alignment, and hg38 with the original annotated gene positions as the target genome for sequencing. Flux Simulator [54] was used to simulate RNA-seq reads from the hg38 genome using Ensembl annotation version 87 [55].

After simulating SVs in the genome, we need to transform the SVs into a set of TSVs, because not all SVs affect the transcriptome, and thus, not all SVs can be detected by RNA-seq. To derive a list of TSVs, we compare the positions of simulated SVs with the gene annotation. If a gene is affected by an SV, some adjacent nucleotides in the corresponding transcript may be in a far part of the RSVsim-generated genome. The adjacent nucleotides may be consecutive nucleotides inside an exon if the breakpoint breaks the exon, or the endpoints of two adjacent exons if the breakpoint hits the intron. So for each SV that hits a gene, we find the pair of nucleotides that are adjacent in the transcript and separated by the breakpoints, and convert them into the coordinates of the RSVsim-generated genome, thus, deriving the TSV.

We compare SQUID to the pipeline of de novo transcriptome assembly and transcript-to-genome alignment. We also use the same set of simulations to test whether existing WGS-based SV detection methods can be directly applied to RNA-seq data. For the de novo transcriptome assembly and transcript-to-genome alignment pipeline, we use all combinations of the existing software Trinity [23], Trans-ABySS [22], GMAP [27], and MUMmer3 [26]. For WGS-based SV detection methods, we test LUMPY [7] and DELLY2 [6]. We test both STAR [56] and SpeedSeq [33] (which is based on BWA-MEM [57]) to align RNA-seq reads to the genome. LUMPY is compatible only with the output of SpeedSeq, so we do not test it with STAR alignments.

## Additional files

Additional file 1 Additional Text, **Table S1** with descriptions, **Figures S1–S8** with descriptions. (PDF 2979 kb)

Additional file 2 Precision and sensitivity table for simulated data with different read lengths and fragment lengths. (TSV 7.43 KB)

Additional file 3 SQUID predictions for the HCC1395 cell line. (TSV 5 KB)

Additional file 4 SQUID predictions for the HCC1954 cell line. (TSV 4 KB)

Additional file 5 Aliquot barcodes of used TCGA samples. (TSV 13 KB)

## Abbreviations

BLCA: Bladder urothelial carcinoma
BRCA: Breast invasive carcinoma
GSG: Genome segment graph
ILP: Integer linear programming
LUAD: Lung adenocarcinoma
PRAD: Prostate adenocarcinoma
SRA: Sequencing Read Archive
SV: Structural variation
TCGA: The Cancer Genome Atlas
TSG: Tumor suppressor gene
TSV: Transcriptomic structural variant
WGS: Whole-genome sequencing
