# Supplementary material for: SQUID: transcriptomic structural variation detection from RNA-seq
Source: Genome Biol. 2018 Apr 12;19:52. doi: 10.1186/s13059-018-1421-5 (PMC5896115; doi:10.1186/s13059-018-1421-5)
Supplement: Supplementary file 1 — Additional Text, Table S1 with descriptions, Figures S1–S8 with descriptions. (PDF 2979 kb) [file 13059_2018_1421_MOESM1_ESM.pdf]

Additional Text

All experiments here are performed with SQUID version 1.3.

Using de novo assembly and transcript to genome alignment to predict TSV

For the pipeline of de novo transcriptome assembly and transcript-to-genome alignment, the direct output is a series of alignment pieces for each assembled transcript. To derive TSV from the pieces of alignment of each transcript, we still need to use the split-read alignment concordance criteria (8) and the edge-building approach. In the case of no TSV, equation (8) still holds, since a transcript is generated from one strand of one chromosome, without rearrangements but only deletion of introns. Any violation of (8) is treated as a TSV. Here TSVs are still able to be represented by edges in GSG, where segments are the intervals of each piece of alignment, and edges are added in the same principle that traversing segments along the edges will result in a concordant alignment of the assembled transcript. The positions of both breakpoints in a TSV are exactly the two positions linked by the discordant edge, and the orientations corresponds to the connection type of the edge.

Processing TCGA RNA-seq data

We use STAR aligner [56] to align TCGA RNA-seq reads to Ensemble genome 87 [53] with the corresponding gene annotation. STAR aligner [56] is set with the option of outputting chimeric alignments with hanging length 15bp. The chimeric alignments generated by STAR [56] are further filtered out if the paired-end reads can be aligned concordantly by SpeedSeq aligner [33]. SQUID is applied to concordant alignments generated by STAR [56] and the filtered chimeric alignments. The discordant edge weight coefficient α is set to be 1, that is, we require tumor transcripts to dominate normal transcripts (if they are incompatible) in order to predict corresponding TSVs.

A large number of fusions between immunoglobulin genes are predicted by SQUID. However, there is possibility that B cells are in the mixture of sequencing and have very high expression of immunoglobulin genes (Ig). We cannot tell whether Ig rearrangements are generated by tumor cells or B cells. Therefore, we exclude Ig TSVs during post-processing and exclude them from the descriptive statistics. Note that SQUID does not exclude Ig TSVs internally, because Ig expression and VDJ recombination have been observed to exist in tumor cells, and revealing the role of Ig in tumors may be useful. When normal cells are removed from tumor samples, using SQUID to predict Ig TSVs may help study relationship between Ig and cancer.

Additional Tables

Table S1: SQUID parameter specification and values in experiments

Symbol	Description	Value
γ	segment degree threshold	4
θ	edge weight threshold	5
α	discordant edge weight coefficient	8 (simulation and HCC cell line), 1 (TCGA)
mq	minimum mapping quality	255 (STAR), 1 (SpeedSeq)
pq	low Phred quality threshold	4 ($p = 10^{-0.4}$)
l	maximum allowed low Phred quality length	10

Note: mq, pq and l are controls for sequencing quality and mapping quality. If mapping quality of a read is less then threshold mq, the read will not be used in edge building. If the read has a low sequencing, in terms of having more than l bases of sequencing quality lower than pq, the read will not be used in edge building.

Additional Figures

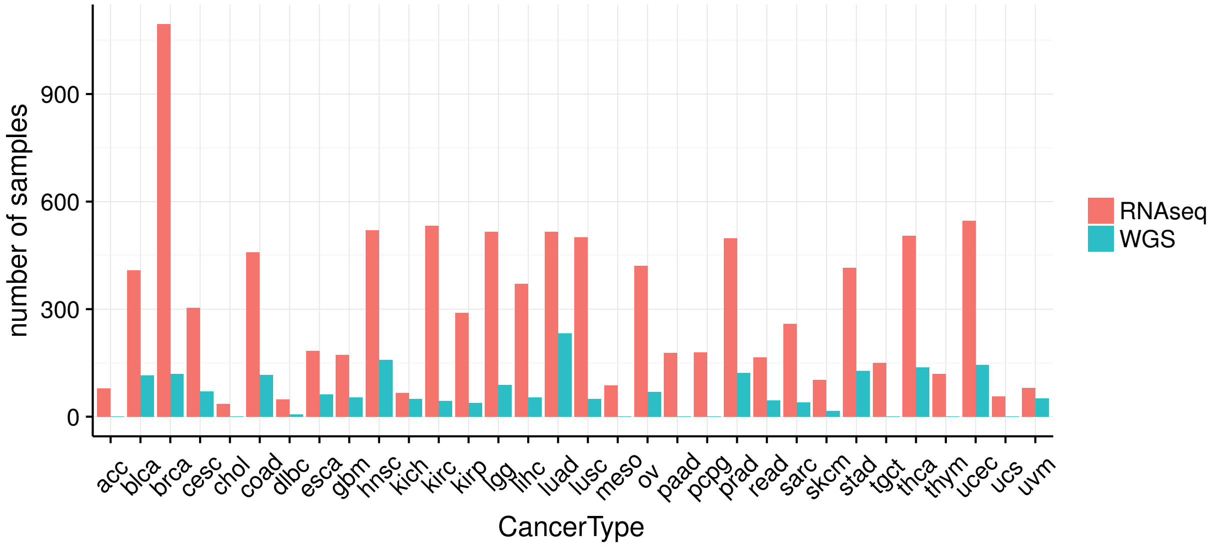

Figure S1: Number of samples with RNA-seq or WGS data in TCGA

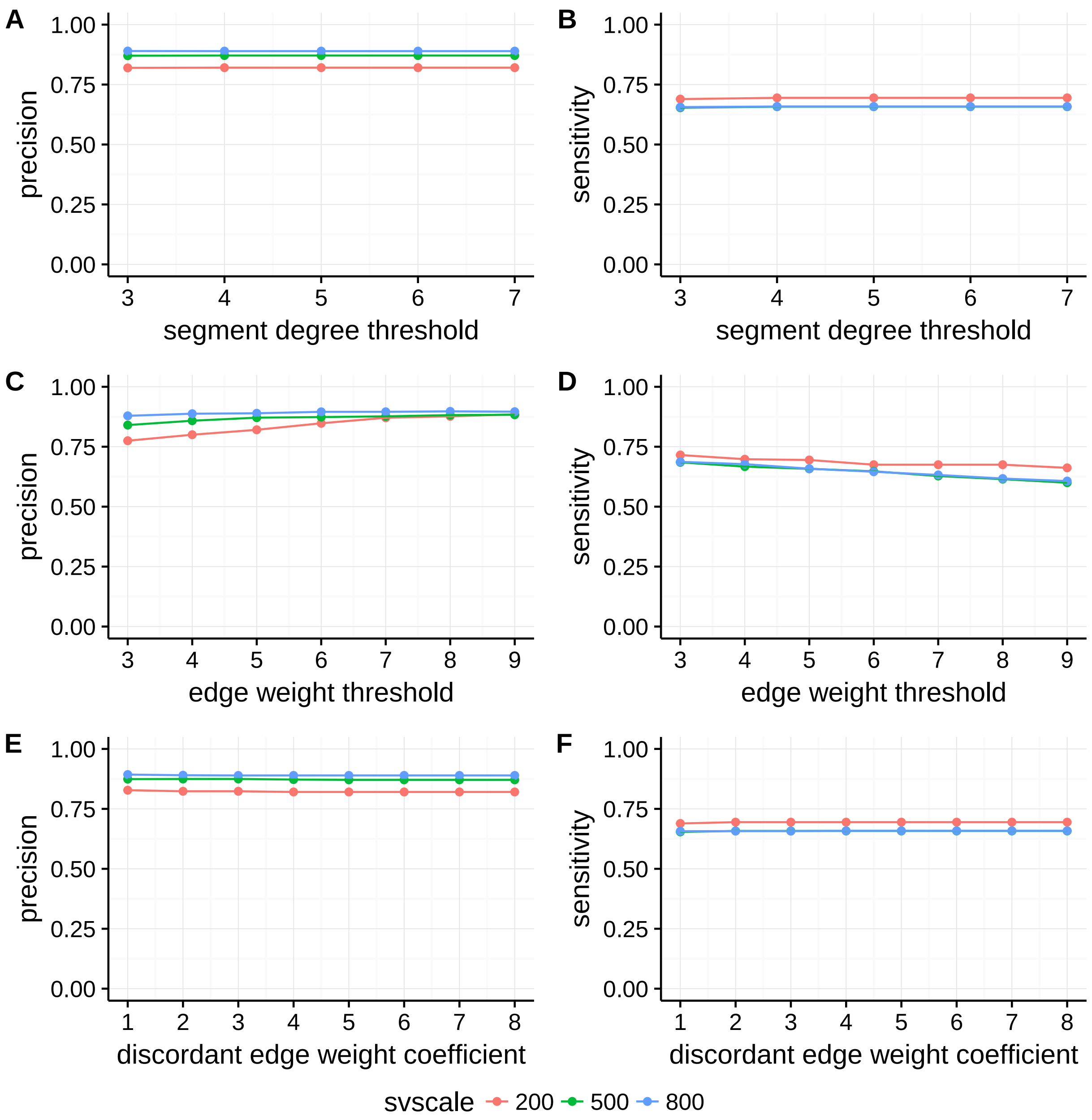

Figure S2: Performance of SQUID on simulation data against different parameters values. (A, B) Segment degree threshold γ. Both the precision and sensitivity curves are relatively flat across different values of γ for all numbers of SVs simulated (200, 500, 800). (C, D) Edge weight threshold θ. Increased value of θ leads to increased precision and decreased sensitivity. This parameter determines the natural precision-sensitivity tradeoff and is one of the most important parameters in SQUID. (E, F) Discordant edge weight coefficient α. This parameter adjusts the edge weights according to normal/tumor cell ratio. Since simulation data is homogeneous, varying this parameter does not change the performance of SQUID.

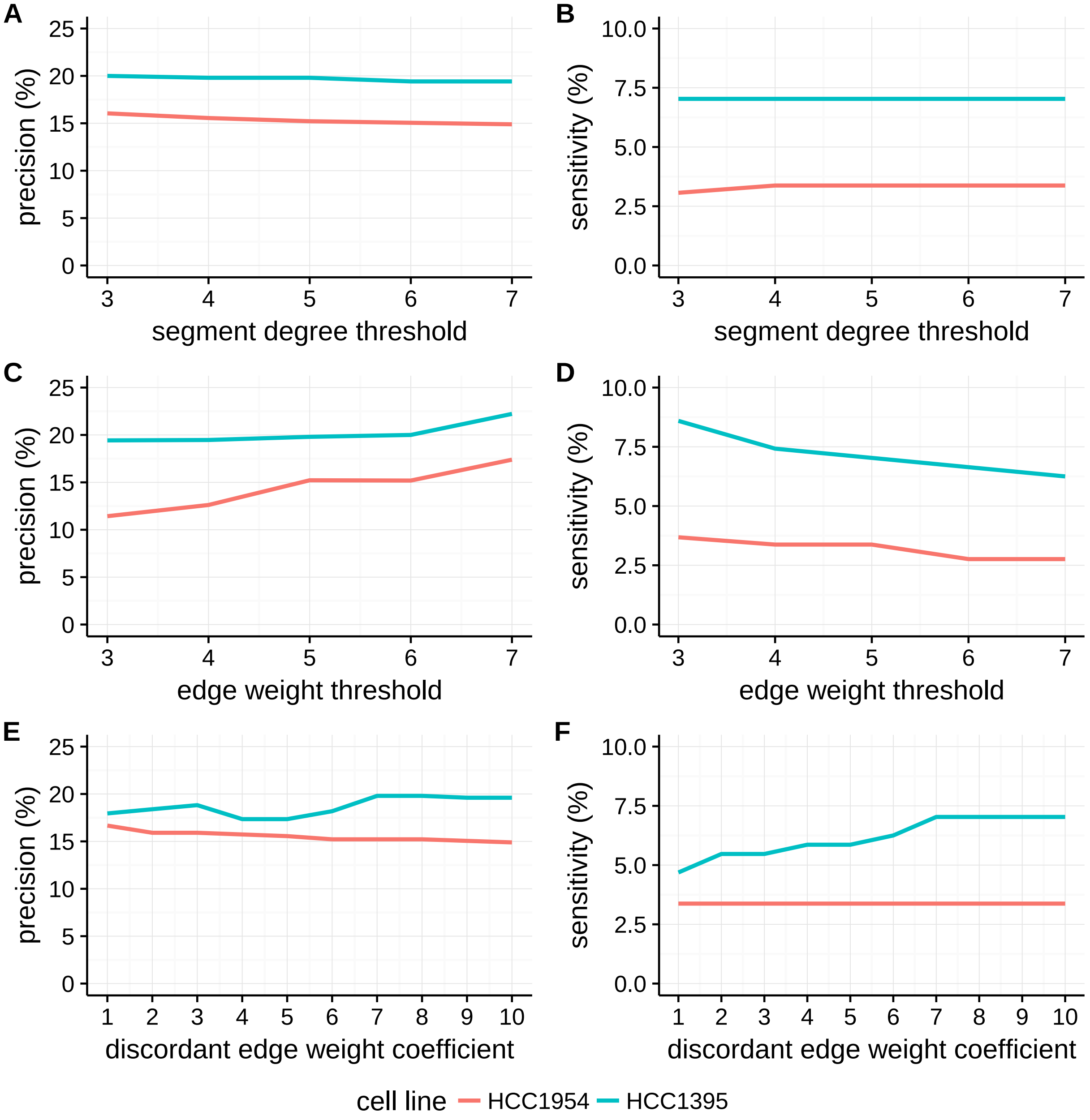

Figure S3: Performance of SQUID on real data against different values of parameters. (A, D) Segment degree threshold γ. Both precision and sensitivity are robust against segment degree threshold. (B, D) Edge weight threshold θ. This parameter affects the natural precision-sensitivity tradeoff. For both HCC1954 and HCC1395 cell lines, increasing θ leads to increased precision and decreased sensitivity. (C, F) Discordant edge weight coefficient α. For HCC1954 cell line, sensitivity does not change when increasing α, indicating rearranged tumor transcripts out-number their normal counterparts; while precision decreases slightly because SQUID predicts more TSVs as discordant edge weight coefficient increases. For HCC1395 cell line, sensitivity and precision reach the highest at discordant edge weight coefficient 8 and remain unchanged at 9 and 10. If some normal transcripts out-number the rearranged tumor transcripts, increasing this parameter allows SQUID to capture these TSVs.

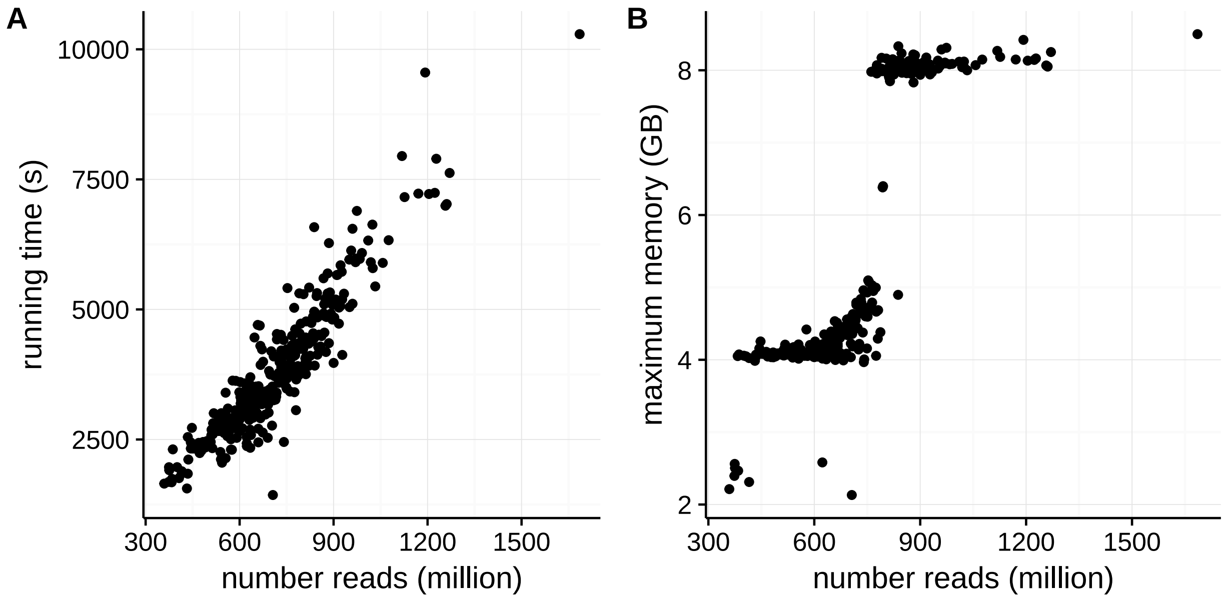

Figure S4: (A) Running time and (B) maximum memory usage of SQUID on TCGA RNA-seq datasets with different number of reads. Alignment time and memory are not included. Running majority of TCGA RNA-seq data takes less than 3 hours, and uses around 4 GB or 8 GB memory.

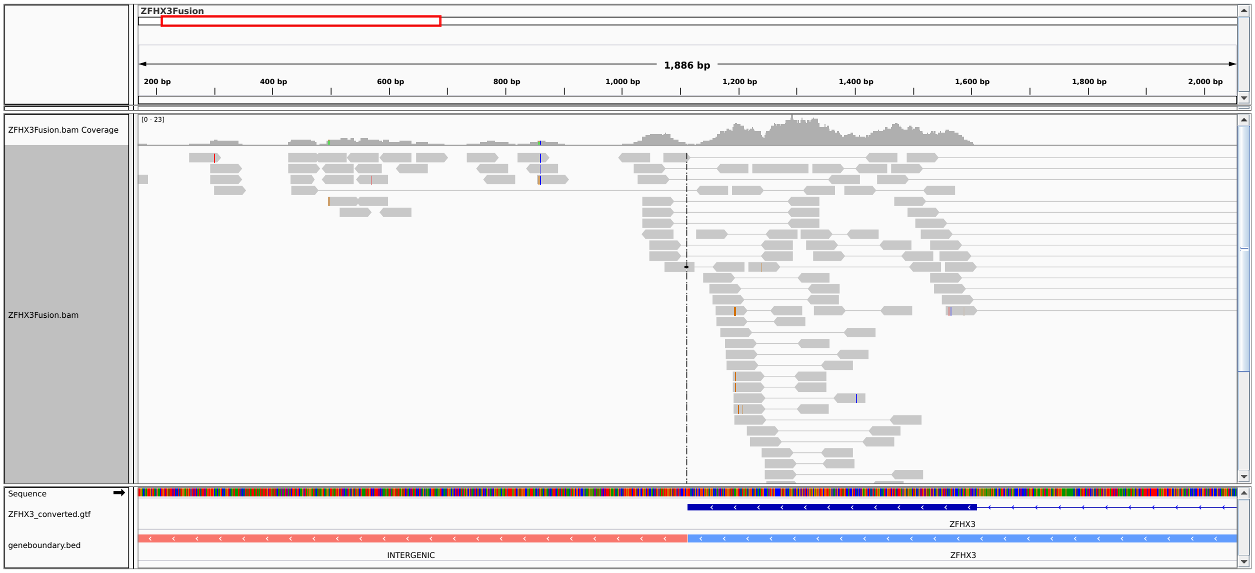

Figure S5: IGV visualization of non-fusion-gene TSV involving *ZFHX3* gene. The reference sequence showed by IGV is the junction sequence of TSV. The first track shows the exons of *ZFHX3* gene in the junction sequence. The second track shows the boundaries of the fused genome segments. In the alignment track, read alignments are viewed as pairs (the grey line links two paired-end alignments). Coverage of intergenic segment is less than coverage of *ZFHX3* gene, which indicates the TSV is heterogeneous and appears in a portion of sequencing sample.

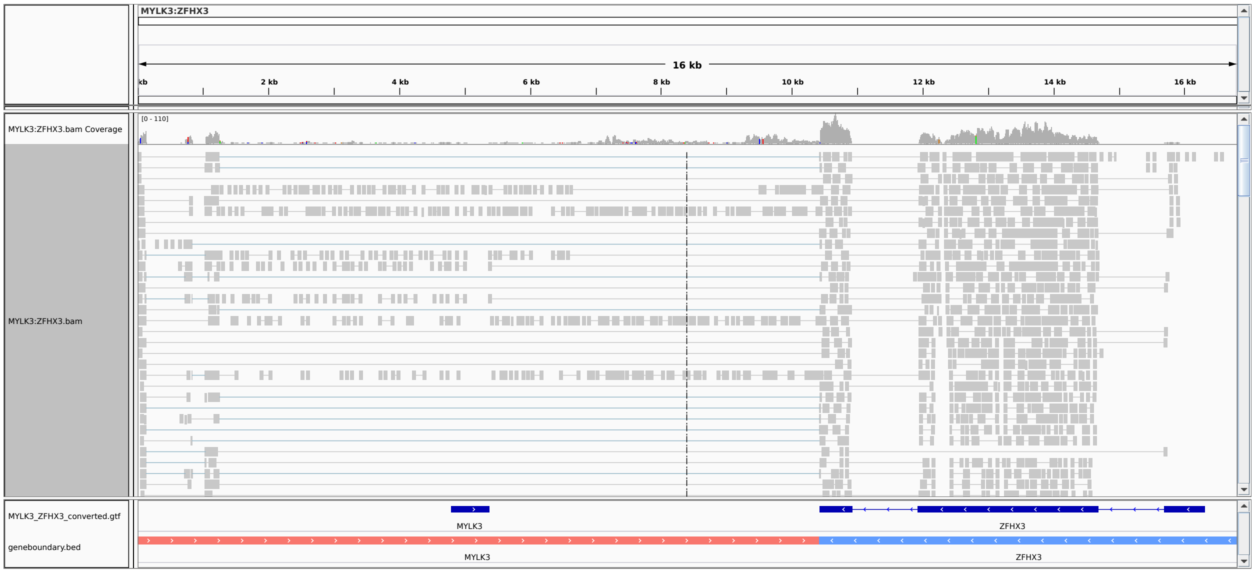

Figure S6: IGV visualization of a non-fusion-gene TSV involving the *ZFHX3* gene and the anti-sense strand of *MYLK3* gene. The reference sequence is the junction sequence of TSV. The first track shows the exons of *ZFHX3* gene and the first exon of *MYLK3* gene in the junction sequence. The second track shows the boundaries of the fused genome segments. In the alignment track, read alignments are viewed as pairs (the grey line links two paired-end alignments, and the blue line links split-read alignments). The large coverage difference between *ZFHX3* gene and anti-sense strand of *MYLK3* gene indicates the TSV is heterogeneous. A splicing event in the segment of *MYLK3* anti-sense strand is indicated by the blue lines in alignment track. The splicing sites do not correspond to the exon of *MYLK3* gene because splicing signals are not preserved on the anti-sense strand. Instead, the new splicing junction is the product of the non-fusion-gene TSV.

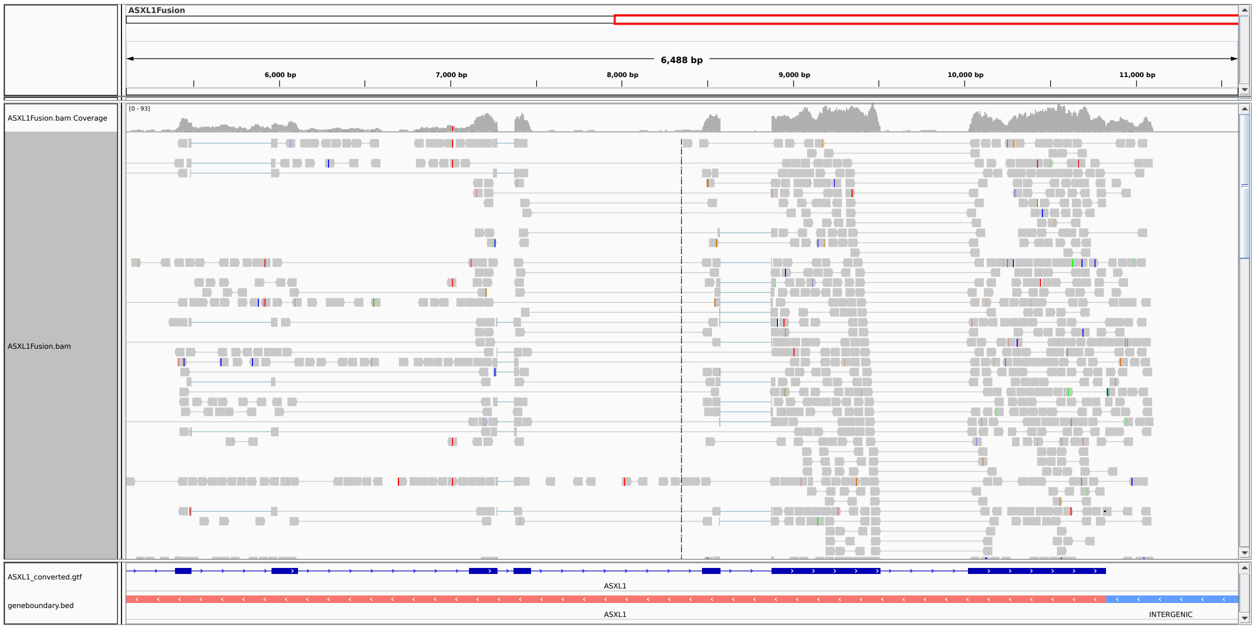

Figure S7: IGV visualization of non-fusion-gene TSV involving *ASXL1* gene. The reference sequence is the junction sequence of TSV. The first track shows the exons of *ASXL1* gene in the junction sequence. The second track shows the boundaries of the fused genome segments. In the alignment track, read alignments are viewed as pairs (the grey line links two paired-end alignments). There are many reads spanning the junction point, and the coverage difference between the two segments is small, which implies the TSV is possibly homogeneous.

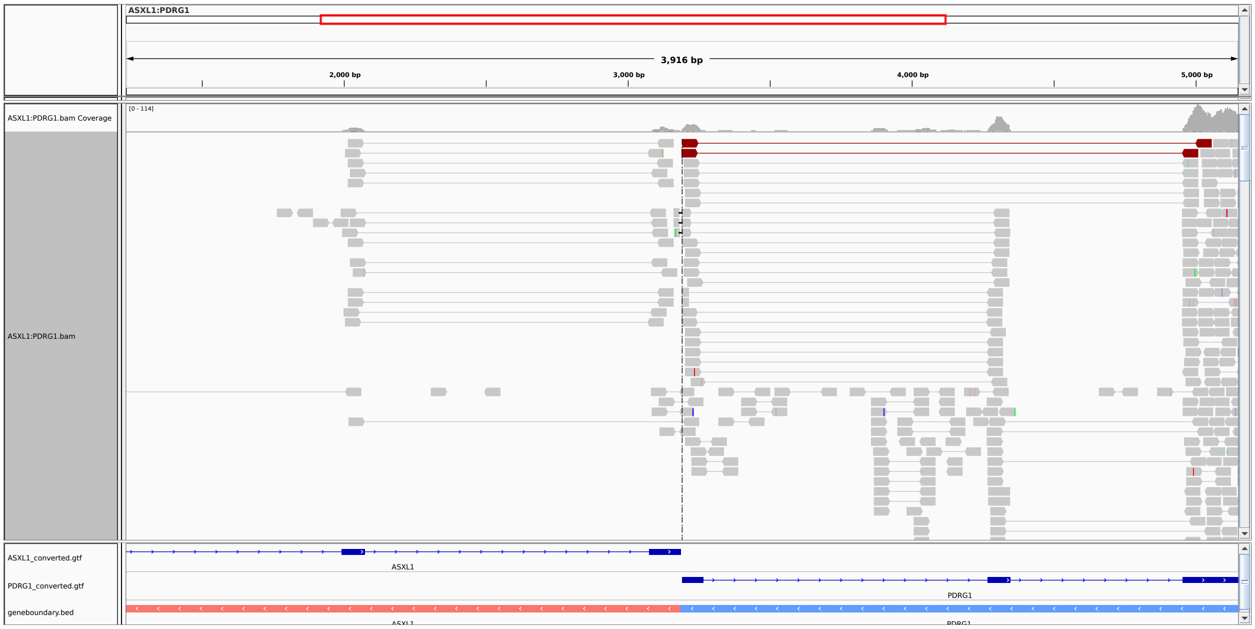

Figure S8: IGV visualization of fusion-gene TSV involving *ASXL1* and *PDRG1* genes. The reference sequence is the junction sequence of TSV. The first two annotation tracks show the exons of *ASXL1* and *PDRG1* gene in the junction sequence. The third track shows the boundaries of the fused genome segments. In the alignment track, read alignments are viewed as pairs (the grey line links two paired-end alignments). There are 8 reads spanning the junction point. The coverage of the *ASXL1* gene is much less than that of the *PDRG1* gene, which implies the fusion-gene TSV is heterogeneous.
